# Cultural Differences in the Relationship Between Negative Leadership Behaviours and Nurses’ Emotional Exhaustion: A Systematic Review

**DOI:** 10.1155/jonm/9748169

**Published:** 2026-03-31

**Authors:** Shiyi Chen, Xinze Zhang, Huan Chen, Feng Zhang, Tao-Hsin Tung, Haixiao Chen

**Affiliations:** ^1^ Evidence-Based Medicine Center, Taizhou Hospital of Zhejiang Province Affiliated to Wenzhou Medical University, Linhai, Zhejiang, China, wmu.edu.cn; ^2^ Cixi Biomedical Research Institute, Wenzhou Medical University, Wenzhou, Zhejiang, China, wmu.edu.cn; ^3^ Department of Orthopedics, Taizhou Hospital of Zhejiang Province Affiliated to Wenzhou Medical University, Linhai, Zhejiang, China, wmu.edu.cn

**Keywords:** abusive supervision, burnout, emotional exhaustion, leadership, negative leadership behaviours, nurses

## Abstract

**Aims:**

Nurses face the risk of emotional exhaustion due to high‐intensity work, resource shortages and multiple stressors. Negative leadership behaviours exacerbate emotional exhaustion through the abuse of power and psychological oppression. Therefore, this study aims to summarise the current evidence on the relationship between negative leadership behaviours and nurse emotional exhaustion in different cultures.

**Methods:**

Databases such as PubMed, Web of Science, Embase, the Cochrane Library, Scopus, CINAHL, APA PsycInfo, CNKI, Wanfang and VIP were searched for related studies, from inception until 20 November 2025, without any language restrictions. The Preferred Reporting Items for Systematic Reviews and Meta‐Analyses (PRISMA) guidelines were used to review these studies. The PEO (population, Exposure and outcome) framework was followed with nurses as the population, negative leadership behaviour as the exposure and emotional exhaustion as the outcome. The final studies were reviewed and selected using EndNote 21, with information extracted according to specific criteria. The Newcastle–Ottawa Scale was used to evaluate the methodological quality of the included studies.

**Results:**

The initial search yielded 1990 records, and after duplicate removal and screening, 11 studies published between 2007 and 2025 were included. Of these, four were longitudinal studies and seven were cross‐sectional studies. Eight of the 11 articles included in this systematic review were conducted in Asia. The correlation coefficients between negative leadership behaviour and emotional exhaustion ranged from 0.18 to 0.76, indicating moderate to strong positive associations, and other variables were found to play a role in the relationship between them.

**Conclusion:**

This systematic review summarises that negative leadership behaviours are significantly positively correlated with nurses’ emotional exhaustion, and this correlation shows significant cultural differences. Future research should conduct cross‐cultural longitudinal studies and develop targeted intervention strategies.

## 1. Background

Healthcare workers experience tremendous pressure due to heavy workloads and high‐intensity work rhythms, making this field highly prevalent for job burnout [1]. Emotional exhaustion, a core dimension of job burnout, is characterised by feelings of exhaustion, excessive stress and depletion of personal resources [2, 3]. Wright and Cropanzano [4] defined emotional exhaustion as ‘a chronic state of physical and emotional depletion caused by excessive work demands and constant trouble’ [4]. Major predictors of emotional exhaustion include excessive workload, lack of social support, work–family conflict and adverse socioeconomic conditions [5]. Previous studies have suggested that emotional exhaustion not only affects the job performance of nurses but may also lead to job burnout, decreased job satisfaction and turnover [6]. In recent years, the medical field has been facing problems such as staff shortages and increased workloads, which further aggravate the risk of emotional exhaustion in nurses [7].

Negative leadership behaviour, which includes abusive supervision, despotic leadership and toxic leadership, occurs when a leader abuses their position of authority in managing staff members, potentially causing physical, psychological, emotional or financial harm [8]. Abusive supervision, the most prevalent manifestation of negative leadership behaviours, is defined as subordinates’ perceptions of the extent to which supervisors engage in sustained hostile verbal and nonverbal behaviours (excluding physical contact) [9] that can directly or indirectly trigger emotional exhaustion [10, 11]. Toxic leadership refers to leaders’ systematic, organised and sustained use of destructive behaviours that inflict psychological harm on followers and damage organisational integrity, with an explicit emphasis on adverse outcomes [12]. For employees, toxic leadership is associated with emotional harm (i.e., fear, emotional exhaustion and social isolation), psychological distress (i.e., depression and anxiety) and physical health problems (i.e., chronic fatigue and insomnia) [13–15].

In addition, despotic leadership emphasises individual dominance and authoritarian conduct, a tendency to prioritise one’s own interests, self‐inflate, and exploiting others to achieve personal goals [16]. As explained by the Conservation of Resources (COR) theory, employees subjected to abusive supervision experience psychological distress due to supervisors withholding critical information [17, 18], leading to the gradual depletion of their psychological and emotional resources and ultimately triggering emotional exhaustion [11, 19]. Complementarily, The Job Demands–Resources (JD–R) model categorises workplace factors into job demands (aspects requiring sustained effort) and job resources (aspects aiding goal achievement) [20]. Within this framework, negative leadership constitutes a potent psychosocial demand that increases strain while simultaneously eroding critical job resources such as supervisor support and autonomy. Leadership behaviour in hospitals is increasingly viewed as a key stressor that can hinder or contribute to work‐related stress and affect staff well‐being [21]. Some studies have shown that emotional exhaustion is a mediator of the negative effects of leadership behaviour on job performance, including cyberloafing [22], counterproductive work behaviours (CWBs) [23] and turnover intentions [10, 24].

It is worth noting that cultural context plays a crucial role in influencing the relationship between leadership behaviour and employee emotional exhaustion. Asian cultures, which typically emphasise hierarchy, collectivism and high power distance, may make negative leadership behaviours (such as authoritarian leadership or abusive supervision) more acceptable or tacitly accepted, thus exacerbating their negative effects [25]. In contrast, Western cultures tend to be more individualistic and egalitarian, employees have less tolerance for inappropriate leadership and organisations often have better feedback and checks and balances [26, 27]. Studies have shown that negative leadership behaviours may lead to higher levels of emotional exhaustion in Asian nursing settings, in part due to increased emotional labour burden due to cultural norms that inhibit direct challenge of authority by staff [28]. In addition, cross‐cultural studies point out that the expression and perception of emotional exhaustion may also differ across cultures, further complicating the relationship between leadership behaviour and emotional well‐being [26]. Therefore, cultural disparity needs to be considered when exploring the relationship between negative leadership behaviours and emotional exhaustion.

Although existing systematic reviews have extensively examined the relationship between leadership behaviours and nurse burnout [29], several critical limitations remain. As highlighted by Niinihuhta and Häggman‐Laitila [29], destructive leadership styles (e.g., exploitative, laissez‐faire) remain understudied in nursing populations, necessitating further research to elucidate the associations between these negative leadership behaviours (including but not limited to destructive leadership) and adverse outcomes. Current reviews typically analyse burnout as a unitary construct, failing to distinguish the unique role of its core dimension—emotional exhaustion. As the most sensitive and earliest manifesting component of burnout syndrome, emotional exhaustion directly reflects an individual’s acute stress response to workplace pressures [30]. Furthermore, previous research indicates that other burnout dimensions—depersonalisation and reduced personal accomplishment—represent Western‐centric concepts that lack cross‐cultural generalisability, whereas emotional exhaustion demonstrates universal relevance [31, 32]. Finally, the high demand for emotional labour in the medical profession makes emotional exhaustion particularly common, but no research has systematically summarised the impact of negative leadership behaviours on emotional exhaustion in this specific dimension, especially the differences in cross‐cultural contexts. This systematic review aims to summarise the relationship between negative leadership behaviours and emotional exhaustion of nurses in different cultures, (1) map associations between negative leadership and emotional exhaustion, (2) examine cultural differences and (3) identify moderators/mediators so as to provide a basis for formulating culturally adaptive leadership intervention strategies.

## 2. Methods

### 2.1. Design

The results of the systematic review are presented following the guidelines outlined in the Preferred Reporting Items for Systematic Reviews and Meta‐Analyses (PRISMA) statement [33]. The study protocol was prospectively registered with PROSPERO on 10 April 2025 (CRD420251028207). Available from https://www.crd.york.ac.uk/PROSPERO/view/CRD420251028207. No deviations from the registered protocol occurred.

### 2.2. Search Methods

Two independent researchers (S.C. and X.Z.) conducted a thorough search for articles published from inception to 20 November 2025 across the following databases: PubMed, Web of Science, Embase, the Cochrane Library, Scopus, CINAHL, APA PsycInfo, CNKI, Wanfang and VIP, to find studies that examined the relationship between negative leadership behaviours and emotional exhaustion among nurses. The combination of five databases—PubMed, Web of Science, Embase, the Cochrane Library and Scopus—can cover literature related to nursing, biomedicine and healthcare management [34]. APA PsycInfo covers all psychological‐related content related to burnout, which may be published in psychological journals. CINAHL was included as the premier nursing‐specific database to ensure comprehensive coverage of nursing workforce studies. Three Chinese databases (CNKI, Wanfang and VIP) are also included to fully capture relevant research in the Chinese medical environment. The search strategy was meticulously developed and applied the PEO framework to ensure the acquisition of all relevant studies [35]. The target population (P) comprised nurses, the exposure (E) focused on negative leadership behaviours and the outcome (O) measured emotional exhaustion. The search strategy involved a combination of relevant MeSH terms and other key terms using Boolean operators (AND, OR and NOT) such as ‘abusive supervision’, ‘negative leadership’, ‘passive leadership’, ‘toxic leadership’, ‘destructive leadership’, ‘exploitative leadership’, ‘intrusive leadership’, ‘despotic leadership’, ‘autocratic leadership’, ‘paternalistic leadership’, ‘unethical leadership’, ‘nonphysical abuse’, ‘narcissistic leadership’, ‘dark side leadership’, ‘adverse leadership’, ‘authoritarian leadership’, ‘laissez‐faire leadership’, ‘emotional exhaustion’, ‘emotional’, ‘job burnout’, ‘burnout’, ‘psychological fatigue’, ‘emotional depletion’, ‘nurse’ and ‘nursing’. To ensure that no articles were overlooked, the reference lists of the retrieved studies were thoroughly searched. The complete search strategy is presented in Table 1.

**TABLE 1:** Search strategy (search date: 2025/11/20).

Source	Search strategies
PubMed (*n* = 993)	#1 ((((((((((((((((((Leadership[MeSH Terms]) OR (Abusive Supervision)) OR (passive leadership)) OR (negative leadership)) OR (destructive leadership)) OR (toxic leadership)) OR (exploitative leadership)) OR (intrusive leadership)) OR (despotic leadership)) OR (autocratic leadership)) OR (paternalistic leadership)) OR (unethical leadership)) OR (nonphysical abuse)) OR (narcissistic leadership)) OR (dark side leadership)) OR (adverse leadership)) OR (authoritarian leadership)) OR (laissez‐faire leadership))) *n* = 110,369 #2 (((((((emotional exhaustion[MeSH Terms]) OR (Emotional Exhaustion[Title/Abstract])) OR (Emotional[Title/Abstract])) OR (Job burnout[Title/Abstract])) OR (burnout[MeSH Terms])) OR (Psychological Fatigue[Title/Abstract])) OR (Emotional Depletion[Title/Abstract])) *n* = 265,349 #3 ((((nurses[MeSH Terms]) OR (nurse)) OR (nursing)) *n* = 1,173,277 #4 #1AND #2 AND #3 *n* = 993

Web of science (*n* = 437)	#1 ((((((((((((((((ALL = (Abusive Supervision)) OR ALL = (passive leadership)) OR ALL = (negative leadership)) OR ALL = (destructive leadership)) OR ALL = (toxic leadership)) OR ALL = (exploitative leadership)) OR ALL = (intrusive leadership)) OR ALL = (despotic leadership)) OR ALL = (autocratic leadership)) OR ALL = (paternalistic leadership)) OR ALL = (unethical leadership)) OR ALL = (nonphysical abuse)) OR ALL = (narcissistic leadership)) OR ALL = (dark side leadership)) OR ALL = (adverse leadership)) OR ALL = (authoritarian leadership)) OR ALL = (laissez‐faire leadership) and Preprint Citation Index (Exclude – Database) and Research Commons (Exclude – Database) *n* = 27,280 #2 (((((TS = (Emotional)) OR TS = (Emotional Exhaustion)) OR TS = (Job burnout)) OR TS = (burnout)) OR TS = (Psychological Fatigue)) OR TS = (Emotional Depletion) and Preprint Citation Index (Exclude – Database) and Research Commons (Exclude – Database) *n* = 537,742 #3 (TS = (nurse)) OR TS = (nursing) and Preprint Citation Index (Exclude – Database) and Research Commons (Exclude – Database) *n* = 1,067,031 #4 #1AND #2 AND #3 *n* = 437

Embase (*n* = 136)	#1 abusive supervision: ti, ab, kw OR passive leadership: ti, ab, kw OR negative leadership: ti, ab, kw OR destructive leadership: ti, ab, kw OR toxic leadership: ti, ab, kw OR exploitative leadership: ti, ab, kw OR intrusive leadership: ti, ab, kw OR despotic leadership: ti, ab, kw OR autocratic leadership: ti, ab, kw OR paternalistic leadership: ti, ab, kw OR unethical leadership: ti, ab, kw OR nonphysical abuse: ti, ab, kw OR narcissistic leadership: ti, ab, kw OR dark side leadership: ti, ab, kw OR adverse leadership: ti, ab, kw OR authoritarian leadership: ti, ab, kw OR laissez‐faire leadership: ti, ab, kw *n* = 7043 #2 emotional exhaustion: ti, ab, kw OR emotional: ti, ab, kw OR job burnout: ti, ab, kw OR burnout: ti, ab, kw OR psychological fatigue: ti, ab, kw OR emotional depletion: ti, ab, kw *n* = 383,580 #3 nursing: ti, ab, kw OR nurse: ti, ab, kw *n* = 559,457 #4 #1AND #2 AND #3 *n* = 136

The Cochrane Library (*n* = 8)	#1 (abusive supervision): ti, ab, kw OR (passive leadership): ti, ab, kw OR (negative leadership): ti, ab, kw OR (destructive leadership): ti, ab, kw OR (toxic leadership): ti, ab, kw OR (exploitative leadership): ti, ab, kw OR (intrusive leadership): ti, ab, kw OR (despotic leadership): ti, ab, kw OR (autocratic leadership): ti, ab, kw OR (paternalistic leadership): ti, ab, kw OR (unethical leadership): ti, ab, kw OR (nonphysical abuse): ti, ab, kw OR (narcissistic leadership): ti, ab, kw OR (dark side leadership): ti, ab, kw OR (adverse leadership): ti, ab, kw OR (authoritarian leadership): ti, ab, kw OR (laissez‐faire leadership): ti, ab, kw *n* = 777 #2 (emotional exhaustion): ti, ab, kw OR (emotional): ti, ab, kw OR (job burnout): ti, ab, kw OR (burnout): ti, ab, kw OR (psychological fatigue): ti, ab, kw OR (emotional depletion): ti, ab, kw *n* = 38,764 #3 (nursing): ti, ab, kw OR (nurse): ti, ab, kw *n* = 56,754 #4 #1AND #2 AND #3 *n* = 8

Scopus (*n* = 268)	#1 ((TITLE‐ABS‐KEY (Abusive Supervision) OR TITLE‐ABS‐KEY (passive leadership) OR TITLE‐ABS‐KEY (negative leadership) OR TITLE‐ABS‐KEY (destructive leadership) OR TITLE‐ABS‐KEY (toxic leadership) OR TITLE‐ABS‐KEY (exploitative leadership) OR TITLE‐ABS‐KEY (intrusive leadership) OR TITLE‐ABS‐KEY (despotic leadership) OR TITLE‐ABS‐KEY (autocratic leadership) OR TITLE‐ABS‐KEY (paternalistic leadership) OR TITLE‐ABS‐KEY (unethical leadership) OR TITLE‐ABS‐KEY (nonphysical abuse) OR TITLE‐ABS‐KEY (narcissistic leadership) OR TITLE‐ABS‐KEY (dark side leadership) OR TITLE‐ABS‐KEY (adverse leadership) OR TITLE‐ABS‐KEY (authoritarian leadership) OR TITLE‐ABS‐KEY (laissez‐faire leadership))) *n* = 20,825 #2 ((TITLE‐ABS‐KEY (Emotional) OR TITLE‐ABS‐KEY (Emotional Exhaustion) OR TITLE‐ABS‐KEY (Job burnout) OR TITLE‐ABS‐KEY (burnout) OR TITLE‐ABS‐KEY (Psychological Fatigue) OR TITLE‐ABS‐KEY (Emotional Depletion))) *n* = 643,532 #3 ((TITLE‐ABS‐KEY (nursing) OR TITLE‐ABS‐KEY (nurse))) *n* = 1,036,025 #4 #1AND #2 AND #3 *n* = 268

CINAHL (*n* = 35)	#1 abusive supervision OR passive leadership OR negative leadership OR destructive leadership OR toxic leadership OR exploitative leadership OR intrusive leadership OR despotic leadership OR autocratic leadership OR paternalistic leadership OR unethical leadership OR nonphysical abuse OR narcissistic leadership OR dark side leadership OR adverse leadership OR authoritarian leadership OR laissez‐faire leadership *n* = 501 #2 emotional exhaustion OR emotional OR job burnout OR burnout OR psychological fatigue1 OR emotional depletion *n* = 134,193 #3 nursing OR nurse *n* = 1,077,775 #4 #1AND #2 AND #3 *n* = 35

APA PsycInfo (*n* = 31)	#1 abusive supervision OR passive leadership OR negative leadership OR destructive leadership OR toxic leadership OR exploitative leadership OR intrusive leadership OR despotic leadership OR autocratic leadership OR paternalistic leadership OR unethical leadership OR nonphysical abuse OR narcissistic leadership OR dark side leadership OR adverse leadership OR authoritarian leadership OR laissez‐faire leadership *n* = 3826 #2 emotional exhaustion OR emotional OR job burnout OR burnout OR psychological fatigue1 OR emotional depletion *n* = 410,744 #3 nursing OR nurse *n* = 225,546 #4 #1AND #2 AND #3 *n* = 31

CNKI (*n* = 26)	#1 TKA = (abusive supervision + passive leadership + negative leadership + destructive leadership + toxic leadership + exploitative leadership + intrusive leadership + despotic leadership + autocratic leadership + paternalistic leadership + unethical leadership + nonphysical abuse + narcissistic leadership + dark side leadership + adverse leadership + authoritarian leadership + laissez‐faire leadership) *n* = 7481 #2 TKA = (emotional exhaustion + emotional + job burnout + burnout + psychological fatigue + emotional depletion) *n* = 766,450 #3 TKA = (nursing + nurse) *n* = 873,850 #4 #1AND #2 AND #3 *n* = 26

Wanfang (*n* = 25)	#1 all Fields: (abusive supervision OR passive leadership OR negative leadership OR destructive leadership OR toxic leadership OR exploitative leadership OR intrusive leadership OR despotic leadership OR autocratic leadership OR paternalistic leadership OR unethical leadership OR nonphysical abuse OR narcissistic leadership OR dark side leadership OR adverse leadership OR authoritarian leadership OR laissez‐faire leadership) *n* = 8679 #2 Title/Abstract: (emotional exhaustion OR emotional OR job burnout OR burnout OR psychological fatigue OR emotional depletion) *n* = 104,223 #3 Title/Abstract: (nursing OR nurse) *n* = 476,582 #4 #1AND #2 AND #3 *n* = 25

VIP (*n* = 31)	#1 T = (abusive supervision OR passive leadership OR negative leadership OR destructive leadership OR toxic leadership OR exploitative leadership OR intrusive leadership OR despotic leadership OR autocratic leadership OR paternalistic leadership OR unethical leadership OR nonphysical abuse OR narcissistic leadership OR dark side leadership OR adverse leadership OR authoritarian leadership OR laissez‐faire leadership) *n* = 10,746 #2 T = (emotional exhaustion OR emotional OR job burnout OR burnout OR psychological fatigue OR emotional depletion) *n* = 47,045 #3 T = (nursing OR nurse) *n* = 273,403 #4 #1AND #2 AND #3 *n* = 31

### 2.3. Eligibility Criteria

Based on the PEO framework, the specific inclusion criteria were defined as follows: (1) observational studies (including cross‐sectional, case–control and cohort studies) and (2) involved registered nurses and nursing assistants. Studies involving mixed healthcare professional populations (e.g., physicians, therapists and nurses) were included only if the results for the nursing subgroup were reported separately and could be extracted. If not, the study was excluded. (3) studies assessing negative leadership behaviours and emotional exhaustion, (4) studies published in peer‐reviewed journals and (5) no language restriction. We excluded qualitative studies, systematic reviews, meta‐analyses, expert opinions and editorials. Unpublished data and grey literature such as papers and conference abstracts were also excluded. These sources are typically not peer‐reviewed, may present incomplete or preliminary data, and may be less consistently available for full‐text review, which can compromise reproducibility and introduce bias.

### 2.4. Study Selection and Data Extraction

The studies found in the databases were entered into the EndNote 21 reference manager software. At first, duplicate studies were removed using the software. Two researchers (C.S.Y. and Z.X.Z.) found and eliminated duplicate entries and then independently screened the retrieved articles based on titles and abstracts to eliminate those irrelevant to the research question, aiming to minimise bias in the data obtained from individuals. Finally, the full‐text articles were searched using the inclusion and exclusion criteria. During the entire process, if there was any disagreement, a third researcher (T‐H.T.) adjudicated until a consensus was reached. To assess the interrater reliability between the two reviewers, we used the kappa statistic to determine consistent study selection and data extraction. The results revealed that the kappa value for data extraction was 0.845 (95% confidence interval (CI): 0.694–0.996). Non‐English articles were translated using machine translation tools (DeepL Translate) for assessment.

A standardised data extraction form was developed and piloted on a sample of 3–5 included studies by the two researchers (C.S.Y. and Z.X.Z.). The form was refined iteratively to ensure clarity and consistency before its formal application to all included studies. Using the finalised form, the two researchers (C.S.Y. and Z.X.Z.) then independently extracted data from all included studies. The authors’ names, year of publication, country, study design, sample size, participant characteristics, type of negative leadership, negative leadership measurement tool, emotional exhaustion measurement tool and findings regarding the relationship between negative leadership and emotional exhaustion in healthcare workers were gathered from the included studies and organised into a structured table.

### 2.5. Quality Appraisal

Two researchers (C.S.Y. and Z.X.Z.) independently evaluated the quality of the literature. The Newcastle–Ottawa Scale (NOS) [36] was used as an identification index to measure the quality of the included literature. In case of discrepancies during the evaluation process, a third trained investigator (TTH) negotiated to solve the problem. The NOS scale was designed in different versions for cross‐sectional and longitudinal studies. Longitudinal studies were evaluated with the original NOS, which allocates up to nine stars across three domains: Selection (representativeness of the exposed cohort, selection of the non‐exposed, ascertainment of exposure, demonstration that outcome was not present at baseline); comparability (control for important confounders); and outcome (assessment method, length and adequacy of follow‐up). Cross‐sectional studies were assessed with the validated NOS adaptation developed by Herzog et al. [37]. This version retains the three‐domain structure but re‐phrases the items to suit single‐time‐point designs: Selection (representativeness, sample‐size justification, handling of non‐respondents, ascertainment of exposure, blinding of assessors), comparability (confounder control) and outcome (validity of outcome measure, appropriateness of statistical test, response rate). Of these, NOS scores ranged from 0 to 10 for cross‐sectional studies and from 0 to 9 for longitudinal studies [38]. The NOS measured quality based on three aspects: selection, comparability and outcomes. Studies with a score of ≥ 7 stars were considered to be of high quality [39].

### 2.6. Data Synthesis

It is very difficult to conduct meta‐analysis due to significant heterogeneity in the included studies. The primary sources of heterogeneity that precluded quantitative pooling were that the exposure variables included conceptually different leadership types (e.g., abusive supervision, toxic leadership, authoritarian leadership and permissive leadership), each with a different theoretical basis and behavioural indicators. There were significant differences in instruments and scales for measuring negative leadership behaviour and emotional exhaustion. And the included studies reported relationships between variables using different statistical measures, which are not easily combined. Therefore, a structured narrative synthesis was conducted following relevant items of the Synthesis without Meta‐analysis (SWiM) guideline to ensure transparent reporting [40]:

1. Grouped studies for synthesis
 To address the primary aim of examining cultural disparity, studies were grouped by their cultural context. Within these cultural groups, studies were further categorised by the type of negative leadership behaviour examined (e.g., abusive supervision, toxic leadership, despotic leadership) to allow for comparisons across different leadership constructs.
2. Defined the synthesis metric and handling measurement heterogeneity
 The correlation coefficient (r) was selected as the standardised metric to summarise the relationship between negative leadership and emotional exhaustion across studies. To transparently address and document the expected measurement heterogeneity—particularly in the scales used to assess emotional exhaustion—we planned to create a comprehensive table detailing all measurement instruments, their scoring ranges and key parameters.
3. Synthesised the characteristics and findings
 For each defined group, we planned to (a) tabulate study characteristics, measurement details and key quantitative findings, (b) narratively summarise the range and distribution of correlation coefficients while considering the methodological quality (NOS scores) of the contributing studies and (c) systematically identify and report any moderating variables or contextual factors described in the primary studies that influenced the relationship of interest. As per the SWiM guidance for narrative synthesis, study quality was thus integrated by qualifying the strength of findings and informing the interpretation of heterogeneity, rather than through sensitivity analysis or weighting.

## 3. Results

### 3.1. Study Selection

**FIGURE 1:**
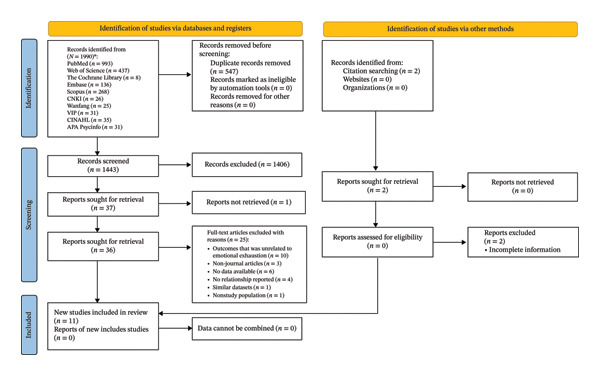
PRISMA flow diagram.

A total of 1990 articles were identified from the 10 databases. After excluding duplicate articles (*n* = 547), 1443 articles were left to be further evaluated for inclusion in the systematic review. Of these, 1406 articles were excluded after screening the titles and abstracts. Finally, 37 articles were selected for full‐text review. After a detailed evaluation, 25 articles were excluded for the following reasons: outcomes unrelated to emotional exhaustion (*n* = 10), no available or extractable data (*n* = 6), no reported relationship between negative leadership and emotional exhaustion (*n* = 4), non‐journal articles (*n* = 3), non‐study population (*n* = 1) and similar datasets (*n* = 1). Consequently, 11 studies met all inclusion criteria and were formally included in the systematic review. The research selection procedure, according to the PRISMA guidelines, is shown in the flowchart in Figure 1.

### 3.2. Characteristics of the Included Studies

Table 2 presents the characteristics of the included studies. The extracted information included the first author, country, year of publication, study design, sample size, sex, age, education, marital status and seniority. A total of 11 studies were published from 2007 to 2025, of which seven studies were published between 2022 and 2024. Except for four longitudinal studies, all the others were cross‐sectional studies. Eight studies were conducted in Asia: three in Pakistan [11, 28, 41], one in China [42], two in Turkey [43, 44], one in Saudi Arabia [45] and one in Iran [46]. The research by Ahmed et al. [13] was conducted in Africa, while the research by Kanste et al. [47] was carried out in Europe. Finally, the research conducted by Whitman et al. [48] was from North America.

**TABLE 2:** Main characteristics of the included studies in the systematic review.

Nos.	Study	Study design	Sample size	Gender	Age (years)	Education (%)	Marital status	Work seniority (years)	Sensitivity analysis, weighting	Weighting
1	[44], Turkey	Cross‐sectional	133	11 males and 122 females	28–35 (32%)	Bachelor and above (82%) Other (18%)	Married (73%) Other (27%)	5–10 (62%)	no	no

2	[11], Pakistan	Two‐wave cross‐lagged panel design	331	62 males and 269 females	30.63 ± 5.09	Nursing diploma (61.6%) Bachelor of science in nursing (BSN) (29.6) Master of Science in nursing (MSN) (8.8%)	NA	7.21 ± 3.59	no	no

3	[13], Egyptian	Cross‐sectional	243	74 males and 169 females	< 30 (58.4%) ≥ 30 (41.6%)	Diploma (7.8%) Technical institute (45.3%) Bachelor’s degree (42.8%) Master’s degree (4.1%)	Married (51.9%) Unmarried (34.6%) Divorced/Widowed (13.6%)	> 10 (70.4%) ≤ 10 (29.6%)	no	no

4	[28], Pakistan	Two‐wave time‐lagged design	225	45 males and 180 females	26–30 (65%)	graduate degrees (75%)	Married (82%)	NA	no	no

5	[42], China	Cross‐sectional	266	13 males and 253 females	18–50	Vocational high school diploma (6.02%) Associate degree (34.21%) Bachelor and above (56.77%) Other (3.01%)	Married (58.27%) Unmarried (40.23%) Divorced/Widowed (1.50%)	1–42	no	no

6	[41], Pakistan	Three‐stage longitudinal design	731	404 males and 327 females	22–26 (32.1%) 26–30 (26.4%) 30–34 (24.6%) ≥ 30 (16.8%)	Two years’ degree (39%) Bachelor’s degree (41.9%) Master’s degree (19.1%)	NA	1–5 (29.8%) 5–9 (23.3%) 9–13 (32.6%) ≥ 13 (14.4%)	no	no

7	[43], Turkey	Cross‐sectional	216	23 males and 193 females	18–28 (21.3%) 29–39 (31.5%) 40–50 (42.1%) ≥ 51 (5.1%)	undergraduate and graduate education (82.9%) below undergraduate (17.1%)	Married (67.6%) Other (33.4%)	< 5 (23.6%) 6–10 (17.6%) ≥ 11 (58.8%)	no	no

8	[45], Saudi Arabia	Cross‐sectional	166	31 males and 135 females	20–29 (34.9%) 30–39 (44.6%) 40–49 (16.3%) 50–59 (4.2%)	NA	NA	1–5 (43.4%) 6–10 (30.7%) 11–15 (15.7%) 16–20 (7.2%) > 20 (3%)	no	yes

9	[47], Finland	Cross‐sectional	601	34 males and 567 females	< 30 (9%) 30–39 (23%) 40–49 (42%) ≥ 50 (26%)	NA	NA	< 5 (13%) 5–20 (47%) > 20 (40%)	no	no

10	[48], United States	Cross‐lagged panel design	460	35 males and 425 females	44.75 ± 11.62	NA	NA	9.54 ± 9.06	no	no

11	[46], Iran	Cross‐sectional	207	37 males and 170 females	22–33 (70.1%) 34–45 (22.7%) > 45 (7.2%)	NA	Married (56%) Single (44%)	< 2 (16.9%) 2–5 (19.3%) > 5 (63.8%)	no	no

### 3.3. Quality Assessment

The NOS scale assessed the four longitudinal studies, with all studies achieving a score ranging from 7 to 9. Seven cross‐sectional studies were evaluated using a modified version of the NOS, except for the study by Akdoğan et al. [43] that received a score of 6, all of which were scored between 7 and 8 out of 10. Overall, all 11 studies in the systematic review showed solid methodological quality. A detailed quality assessment is shown in Tables 3 and 4. In cross‐sectional studies, the correlation coefficient reported by Akdoğan et al. [43] (*r* = 0.43) was in the middle range of the overall correlation spectrum reported by high‐quality studies (*r* = 0.18–0.76). Longitudinal studies all had a high‐quality score of 7, but the reported effect sizes (*β*) still indicated that the differences in findings could not be attributed to the different quality of this study. Therefore, this implied that there is no effect size bias in relative low‐quality studies.

**TABLE 3:** Quality assessment of cross‐sectional studies assessed using the Newcastle–Ottawa Scale (NOS).

Author, year	1. Selection				2. Comparability	3. Outcome		Total quality scores
	Representativeness of the sample	Sample size	Non‐respondents	Ascertainment of exposure (risk factor)	The subjects in different outcome groups are comparable, based on the study design or analysis. Confounding factors are controlled	Assessment of outcome	Statistical test	
Koç et al., 2022 [44]	1	1	1	2	1	1	1	8
Ahmed et al., 2024 [13]	1	1	0	2	1	1	1	7
Chen et al., [42]	1	1	0	2	1	1	1	7
Akdoğan et al., [43]	0	1	0	2	1	1	1	6
Alsadaan, 2025 [45]	1	1	1	2	1	1	1	8
Kanste et al., [47]	1	1	0	2	1	1	1	7
Ebrahimzade et al., [46]	1	1	0	2	1	1	1	7

*Note:* ≥ 7 points: good quality (low risk of bias); 4–6 points: fair quality (moderate risk of bias); and ≤ 3 points: poor quality (high risk of bias).

**TABLE 4:** Quality assessment of longitudinal studies assessed using the Newcastle–Ottawa Scale (NOS).

Author, year	1. Selection				2. Comparability	3. Outcome			Total quality scores
	Representativeness of the exposed cohort	Selection of the non‐exposed cohort	Ascertainment of exposure	Demonstration that outcome of interest was not present at the start of the study	Comparability of cohorts based on the design or analysis controlled for confounders	Assessment of outcome	Was follow‐up long enough for outcomes to occur	Adequacy of follow‐up of cohorts	
Malik et al., [11]	1	1	1	0	1	1	1	1	7
Rafiq et al., [28]	1	1	1	0	1	1	1	1	7
Badar et al., [41]	1	0	1	1	1	1	1	1	7
Whitman et al., 2012	1	0	1	1	1	1	1	1	7

*Note:* ≥ 7 points: good quality (low risk of bias); 4–6 points: fair quality (moderate risk of bias); and ≤ 3 points: poor quality (high risk of bias).

### 3.4. Negative Leadership Behaviours

Table 5 summaries the measures of negative leadership and emotional exhaustion and the parameters used in the included studies. Three studies [11, 42, 48] focused on abusive supervision, two [13, 44] on toxic leadership, two [28, 43] on despotic leadership, two [46, 47] on laissez‐faire leadership and one [45] study on destructive leadership. In addition, there was a study that focused on both despotic and narcissistic leadership [41]. Abuse supervision scores ranged from 1.89 to 3.98 on average. The average score for nurses’ perceptions of toxic leadership was 3.31 ± 1.09 [13]. The average scores for nurses’ perceptions of despotic leadership were 2.69 ± 0.73, 4.57 ± 1.36 and 2.38 ± 1.08 [28, 41, 43]. The average score for nurses’ perceptions of narcissistic leadership perception was 4.48 ± 1.49 [41]. Furthermore, four studies [44–47] did not report any specific method.

**TABLE 5:** Negative leadership and emotional exhaustion measures with parameters used in the included studies.

Nos.	First author, year	Negative leadership type	Negative leadership scale (range)	Reliability and validity	Negative leadership mean ± SD	Emotional exhaustion scale (range)	Reliability and validity	Cross‐cultural validation	Emotional exhaustion mean ± SD	Sample size	Correlation coefficient (emotional exhaustion and negative leadership)
1	[44], Turkey	toxic leadership	Toxic Leadership Scale [70] (Likert, 1–5)	Cronbach’s *α*: 0.89–0.96 CR: 0.92–0.97 AVE:0.69–0.80	NA	9‐item emotional exhaustion scale is adapted from MBI [3] (Likert, 1–5)	Cronbach’s *α*: 0.95 CR: 0.96 AVE:0.78	NA	NA	133	0.28^∗∗^
2	[11], Pakistan	abusive supervision	Abusive Supervision Scale [71] (Likert, 1–5)	CR: 0.936 (T1) 0.929 (T2) AVE: 0.745 (T1) 0.722 (T2)	3.98 ± 0.81	3‐item emotional exhaustion scale is adapted from MBI [49] (Likert, 1–5)	CR: 0.837 (T1) 0.838 (T2) AVE:0.631 (T1) 0.633 (T2)	NA	3.86 ± 0.73^b^	331	0.54^∗∗^
3	[13], Egyptian	toxic leadership	Toxic Leadership Scale (ToxBH‐N) [14] (Likert, 1–5)	Cronbach’s *α*: 0.88	3.31 ± 1.09	9‐item emotional exhaustion scale (MBI subscale) [82] (Likert, 0–5)	Cronbach’s *α*: 0.76	YES	31.65 ± 7.94^a^	243	0.53^∗∗∗^
4	[28], Pakistan	despotic leadership	Despotic Leadership Scale [16] (Likert, 1–5)	Cronbach’s *α*: 0.89 CR: 0.92 AVE > 0.50	2.69 ± 0.73	3‐item emotional exhaustion scale [50] (Likert, 1–5)	Cronbach’s *α*: 0.82 CR: 0.95 AVE > 0.50	YES	2.95 ± 0.46^b^	225	0.46^∗∗^
5	[42], China	abusive supervision	Abusive Supervision Scale [9] (Likert, 1–7)	Cronbach’s *α*: 0.972	2.44 ± 1.51	5‐item emotional exhaustion scale [72] (Likert, 1–7)	Cronbach’s *α*: 0.886	YES	4.47 ± 1.52^b^	266	0.25^∗∗^
6	[41], Pakistan	despotic leadership	Despotic Leadership Scale [16] (Likert, 1–7)	Cronbach’s *α*: 0.942 CR: 0.954 AVE: 0.776	4.57 ± 1.36	6‐item emotional exhaustion scale [73] (Likert, 1–7)	Cronbach’s *α*: 0.919 CR: 0.942 AVE: 0.804	YES	4.09 ± 1.56^b^	731	0.76^∗∗^
7	[41], Pakistan	narcissistic leadership	Narcissistic Leadership Scale [74] (Likert, 1–7)	Cronbach’s *α*: 0.934 CR: 0.948 AVE: 0.754	4.48 ± 1.49	6‐item emotional exhaustion scale [73] (Likert, 1–7)	Cronbach’s *α*: 0.919 CR: 0.942 AVE: 0.804	YES	4.09 ± 1.56^b^	731	0.74^∗∗^
8	[43], Turkey	despotic leadership	Despotic Leadership Scale [75, 75] (−)	Cronbach’s *α*: 0.946 CR: 0.957 AVE: 0.787	2.38 ± 1.08	5‐item emotional exhaustion scale (MBI subscale) [76] (−)	Cronbach’s *α*: 0.901 CR: 0.924 AVE: 0.710	YES	2.73 ± 0.97^b^	216	0.43^∗∗∗^
9	[45], Saudi Arabia	destructive leadership	Destructive Leadership Questionnaire (DLQ) [77] (Likert, 1–5)	Cronbach’s *α*: 0.88–0.93	NA	MBI‐HSS subscale [78] (Likert, 0–6)	Cronbach’s *α*: 0.71–0.90	YES	NA	166	0.49^∗∗^
10	[48], United States	abusive supervision	Abusive Supervision Scale [79] (Likert, 1–7)	Cronbach’s *α*: 0.97 (T1) 0.94 (T2) 0.95 (T3)	1.89 ± 1.23	5‐item emotional exhaustion scale (MBI‐GS subscale) [78] (Frequency, 1–5)	Cronbach’s *α*: 0.92 (T1) 0.90 (T2) 0.89 (T3)	YES	2.81 ± 0.78^b^	601	0.31^∗∗^
11	[47], Finland	laissez‐faire leadership	Multifactor Leadership Questionnaire (MLQ) [80] (Likert, 1–5)	Cronbach’s *α*: 0.78	NA	6‐item emotional exhaustion scale (MBI‐HSS subscale) [78] (Likert, 1–7)	Cronbach’s *α*: 0.87	YES	NA	460	0.18^∗∗∗^
12	[46], Iran	laissez‐faire leadership	Multifactor Leadership Questionnaire (MLQ) [81] (−)	Cronbach’s *α*: 0.95	NA	9‐item emotional exhaustion scale (MBI subscale) [78] (−)	Cronbach’s *α*: 0.73	YES	27.26^a^	207	−0.005

Abbreviations: AVE, Average Variance Extracted; CR, Composite Scale Reliability; MBI, Maslach Burnout Inventory; MBI‐HSS, Maslach Burnout Inventory‐Human Services Survey; MBI‐GS, Maslach Burnout Inventory–General Survey.

^a^Total scores.

^b^Subscale scores.

^∗∗^ *p* < 0.01.

^∗∗∗^ *p* < 0.001.

### 3.5. Emotional Exhaustion

The measurement of emotional exhaustion across the included studies exhibited notable heterogeneity in scales and scoring conventions. As summarised in Table 5, the Maslach Burnout Inventory (MBI) and its various adaptations were the most common, used in seven studies [13, 43–48], which included different versions (e.g., MBI‐HSS, MBI‐GS) and subscale lengths (ranging from 3 to 9 items). The remaining four studies utilised other validated scales, such as those by Riley et al. [49] and Pines et al. [50]. Of the 11 studies, 9 (81.8%) explicitly reported using scales that had undergone cross‐cultural validation or adaptation for their specific context. The remaining two studies did not report on this aspect. Crucially, the reported emotional exhaustion means are based on fundamentally different metrics (e.g., total MBI scores, MBI emotional exhaustion subscale scores and scores from entirely different instruments). Due to the fundamental differences in the scale construction, validation and scoring algorithms among these various indicators, it is very difficult to directly make comparisons for conversion.

### 3.6. Negative Leadership Behaviours and Emotional Exhaustion Among Nurses

The included studies showed that the correlation coefficient between negative leadership behaviour and emotional exhaustion among nurses were all significant in 10 out of 11 studies, ranging from 0.18 to 0.76, indicating moderate‐to‐strong positive associations. The only non‐significant finding was reported by Ebrahimzade et al. [46], who found no significant relationship between laissez‐faire leadership and emotional exhaustion. Longitudinal evidence, as summarised in Table 6, provides robust support for the temporal precedence and predictive impact of negative leadership on emotional exhaustion. Specifically, Time 1 measures of abusive supervision [11, 48], despotic leadership [28, 41] and narcissistic leadership [41] all significantly predicted higher levels of emotional exhaustion at subsequent time points (Time 2). This consistent pattern across studies strongly indicates that hospital leaders’ negative leadership behaviours exacerbated the emotional exhaustion of nurses.

**TABLE 6:** Longitudinal studies examining the impact of negative leadership styles on emotional exhaustion.

Study (author, year)	Design (waves)	Negative leadership type (time X)	Emotional exhaustion (time Y)	Key longitudinal finding (effect size and direction)
[11]	Longitudinal (T1, T2, 6‐month lag)	Abusive supervision (T1)	Emotional exhaustion (T2)	T1 AS ⟶ T2 EE: *β* = 0.270^∗∗∗^
[28]	Time‐lagged (T1, T2, 2‐week lag)	Despotic leadership (T1)	Emotional exhaustion (T2)	T1 DL ⟶ T2 EE: *β* = 0.290^∗∗∗^
[41]	Three‐wave longitudinal (T1, T2, T3, 2‐week intervals)	Despotic leadership (T1)	Emotional exhaustion (T2)	T1 DL ⟶ T2 EE: *β* = 0.472^∗∗∗^
[41]	Three‐wave longitudinal (T1, T2, T3, 2‐week intervals)	Narcissistic leadership (T1)	Emotional exhaustion (T2)	T1 NARL ⟶ T2 EE: *β* = 0.436^∗∗∗^
[48]	Three‐wave longitudinal (T1, T2, T3, 1‐week intervals)	Abusive supervision (T1)	Emotional exhaustion (T2)	T1 AS ⟶ T2 EE: NA

^∗∗∗^ *p* < 0.001.

Five included articles showed that other variables influence the relationship between negative leadership behaviours and nurses’ emotional exhaustion [11, 28, 42, 44, 47]. Koç et al. [44] showed that intrinsic motivation had a moderating effect on the relationship between toxic leadership and nurses’ emotional exhaustion. Malik et al. [11] found that self‐compassion attenuated the positive cross‐lagged effect of abusive supervision on emotional exhaustion. Rafiq et al. [28] showed that the fear of COVID‐19 positively moderated the effect of despotic leadership on nurses’ emotional exhaustion. Chen et al. [42] found that subordinate ingratiation positively moderated the relationship between abusive supervision and nurses’ emotional exhaustion. Kanste et al. [47] found that the employment status significantly moderated the relationship between laissez‐faire leadership and nurses’ emotional exhaustion.

## 4. Discussion

### 4.1. Clinical Implications

This systematic review found evidence from 11 studies in different cultural backgrounds summarising the relationship between negative leadership behaviours and emotional exhaustion in nurses. While the findings support a positive association, our analysis reveals key nuances related to leadership type, cultural context, moderating variables and methodological limitations that have driven the existing literature.

Previous research has shown that abusive supervision is positively associated with emotional exhaustion [51]. COR theory states that abusive supervision depletes employees’ resources because it might threaten their current work status and reduce psychological resources [17, 52]. When employees are subjected to abusive supervision, they usually suppress the expression of negative emotions towards their supervisors. Emotional regulation consumes a large amount of emotional resources and leads to emotional exhaustion [51]. Studies have proven that emotional exhaustion can undermine employees’ job performance [53, 54]. According to the JD–R model, abusive supervision is a type of obstructive job demand that consumes employees’ energy and leads to emotional exhaustion. Abusive supervision increases the work stress of employees and makes them feel overwhelmed, which triggers emotional exhaustion [11, 20]. Toxic leadership can undermine team morale and trust, produce toxic work conditions and place the leader’s success ahead of the team’s well‐being. This creates stress, affects the health of employees and leads to burnout [13, 44]. Emotional resources are one such resource that can be lost, and they stand out because they direct the use of and help attract other resources. Thus, the loss of emotional resources is significant. Emotional exhaustion occurs when individuals feel that they do not have sufficient resources to deal with the stressors they face, and negative leadership behaviour is seen as an extreme social stressor [41].

Many previous studies have examined the relationship between negative leadership behaviours and emotional exhaustion in companies or organisations outside of healthcare, but few have focused on the healthcare industry [22, 55, 56]. We found that among the studies on negative leadership behaviours and emotional exhaustion of nurses included in the analysis, 8 of the 11 studies were conducted in countries often characterised in cross‐cultural research as having higher levels of power distance (e.g., Pakistan, Turkey, China) [57]. It is important to note that these studies [11, 28, 41] generally reported stronger associations. This pattern may be explained by contextual mechanisms prevalent in such settings, where high power distance can normalise hierarchical authority and constrain organisational grievance pathways [58, 59], potentially amplifying subordinates’ perceived helplessness and resource depletion in the face of negative leadership [25, 28]. In contrast, studies conducted in contexts characterised by lower power distance and more developed institutional safeguards for employees reported weaker correlations [47, 48]. In such settings, factors such as greater career mobility and formalised mechanisms for reporting misconduct may provide employees with more resources to cope with or exit from negative leadership situations, thereby attenuating its impact on emotional exhaustion [26]. In addition, it is crucial to acknowledge heterogeneity within regions; cultural dimensions and organisational practices vary significantly even among countries sharing a broad geographical label (e.g., ‘Asia’). Therefore, the observed differences are more accurately attributed to specific socio‐contextual factors like power distance and organisational grievance pathways, rather than to deterministic cultural categories.

The results showed that the type of negative leadership behaviour significantly moderated the strength of its relationship with emotional exhaustion. Despotic leadership, toxic leadership and abusive supervision are strongly correlated with emotional exhaustion in nurses, which is in line with the resource conservation theory (COR). When the leadership style is destructive and characterised by intimidation, lack of support and excessive criticism, the obvious hostile behaviour of the leader will directly consume the emotional resources of the subordinates, thereby leading to emotional exhaustion [17, 28]. In contrast, laissez‐faire leadership has weak or even no significant correlation with emotional exhaustion of nurses, which may be due to the fact that leaders of this style avoid decision‐making, transfer responsibility and play an independent role in managing the affairs and situations of the organisation, so the effect on emotional resource consumption of subordinates is very limited [46, 47].

Of the 11 included studies, 7 were published between 2022 and 2025, indicating that research on the relationship between negative leadership behaviours and emotional exhaustion among nurses has increased in recent years. The COVID‐19 pandemic, unprecedented workloads, long working hours and fear of contracting the virus have further exacerbated nurses’ stress and emotional exhaustion [60]. For example, the studies included in this study [13, 28] explicitly linked pandemic‐related stressors, including fear of infection, increased workload and organisational instability, to heightened emotional exhaustion under negative leadership [61]. In recent years, there has been increasing global concern about nurse turnover and mental health issues, especially in light of widespread staff shortages [62, 63]. Studies have shown that emotional exhaustion is a predictor of turnover intention in nurses [64]. Studies such as Badar et al. [41] and Alsadaan [45] directly link disruptive leadership styles to turnover intentions, reflecting the urgency to create a safe and supportive work environment that encourages open communication and quick action on negative leadership behaviours, which is critical for nurse well‐being and retention. It is worth noting that studies in recent years have improved in methodology, with three using longitudinal study designs, which can help to better understand the causal relationship between variables.

This study found that the association between negative leadership behaviours and emotional exhaustion was complicated by multiple moderating variables, a finding that deepens the application of COR theory in the context of healthcare organisations. It has been shown that both individual internal resources (e.g., self‐compassion and intrinsic motivation) and external contextual resources (e.g. nurse–patient relationship) can buffer the impact of negative leadership behaviours on emotional exhaustion, which enriches the study of López‐Cabarcos et al. [65] on emotional exhaustion among public healthcare professionals. It is worth noting that the moderating variable may have the characteristics of a double‐edged sword. Although subordinate integrated behaviour may effectively increase leadership deployment relations and relieve leadership pressure in the short term, it may exacerbate emotional exhaustion by reinforcing power imbalance in the long term [42]. Rafiq et al. pointed out that the moderating role of fear in emergency public health situations suggests that crisis environments may amplify the psychological costs of authoritarian leadership [28]. Furthermore, most previous studies have used emotional exhaustion as a mediating variable [10, 11, 13, 22, 28, 41]; however, to determine the direct impact of negative leadership behaviours on emotional exhaustion in healthcare workers, this study included emotional exhaustion as a dependent variable. Second, most previous research on negative leadership behaviours has focused on the corporate environment; however, the current status of negative leadership behaviours in healthcare and their impact on employees’ emotional exhaustion have been less explored. The articles included in this review were all medical studies, which broadened our understanding of negative leadership behaviours and emotional exhaustion. Finally, the research literature on the relationship between negative leadership behaviour and emotional exhaustion in healthcare workers is enhanced by the fact that variables such as intrinsic motivation and self‐compassion play distinct roles in both of these issues [65].

### 4.2. Clinical Practice

The synthesised evidence underscores the necessity to implement multi‐level interventions in healthcare settings. First and foremost, healthcare institutions should implement mandatory, evidence‐based training for all leadership roles (e.g., nurse supervisors, department heads) to enhance awareness of the harms of negative behaviours and cultivate constructive leadership skills, a strategy directly supported by recent evidence [41, 66]. This should be integrated with robust, confidential and multi‐access reporting channels alongside clear, enforced leadership standards to ensure accountability. Concurrently, staff support must be strengthened through evidence‐based measures such as workload optimisation, enhanced professional autonomy and dedicated career development programmes, which are shown to mitigate emotional exhaustion and improve retention [65, 66]. In Asian contexts specifically, we propose that the culturally targeted layer is critical, such as fostering sanctioned peer support networks and organisation‐wide communication campaigns that promote psychological safety. Together, these multi‐level interventions—with established evidence for leadership and staff support components and a proposed cultural adaptation—are expected to reduce burnout, turnover and absenteeism while fostering a sustainable and healthy work environment.

### 4.3. Limitations

This systematic review has some limitations. First, only a few fully eligible studies were included in this study, which may affect the generalisability of our findings to all nursing contexts. This is primarily a function of the highly specific focus of our review. Second, with only four longitudinal studies, it is difficult to determine true causality. Further longitudinal or experimental studies are needed to better elucidate the causal path of this relationship [11]. Third, methodological diversity and cultural adaptation in measuring emotional exhaustion make it difficult to compare studies. Future research strongly recommends the adoption of cross‐culturally validated instruments, such as MBI‐HSS [67, 68]. This tool is widely considered the gold standard for measuring burnout in healthcare workers and has been extensively applied in studies involving nurses across diverse cultural contexts, thereby facilitating future evidence synthesis [45]. Fourth, the selected studies could not be subjected to a meta‐analysis because of the inconsistent information provided. To facilitate quantitative pooling in future meta‐analyses, studies should adhere to minimum reporting standards by consistently providing effect sizes (*β* coefficients) alongside their confidence intervals for key relationships, rather than only significance tests [69]. Finally, only the reported results of the studies, not the original data, were analysed. Future systematic reviews could attempt to obtain individual participant data or original datasets where available to enable more comprehensive and nuanced analyses.

## 5. Conclusions

This systematic review summarises that negative leadership behaviours are significantly positively correlated with nurses’ emotional exhaustion, and this correlation shows significant cultural differences. Future research should conduct cross‐cultural longitudinal studies and develop targeted intervention strategies.

## Author Contributions

Shiyi Chen contributed to conception, design and drafting of the manuscript. Shiyi Chen, Xinze Zhang, Huan Chen and Feng Zhang contributed to acquisition, analysis or interpretation of data. Tao‐Hsin Tung and Haixiao Chen contributed to supervision.

## Funding

This work was supported by the National Natural Science Foundation of China (funding ID 72374157).

## Ethics Statement

The authors have nothing to report.

## Conflicts of Interest

The authors declare no conflicts of interest.

## Data Availability

Data sharing is not applicable to this article as no datasets were generated or analysed during the current study.
